# (p)ppGpp Metabolism and Antimicrobial Resistance in Bacterial Pathogens

**DOI:** 10.3389/fmicb.2020.563944

**Published:** 2020-10-09

**Authors:** Bhabatosh Das, Rupak K. Bhadra

**Affiliations:** ^1^Infection and Immunology Division, Translational Health Science and Technology Institute (THSTI), Faridabad, India; ^2^Infectious Diseases and Immunology Division, Council of Scientific and Industrial Research-Indian Institute of Chemical Biology (CSIR-IICB), Kolkata, India

**Keywords:** bacterial pathogen, antibiotic resistance, *spoT*, *relA*, stringent response, (p)ppGpp, alarmone

## Abstract

Single cell microorganisms including pathogens relentlessly face myriads of physicochemical stresses in their living environment. In order to survive and multiply under such unfavorable conditions, microbes have evolved with complex genetic networks, which allow them to sense and respond against these stresses. Stringent response is one such adaptive mechanism where bacteria can survive under nutrient starvation and other related stresses. The effector molecules for the stringent response are guanosine-5'-triphosphate 3'-diphosphate (pppGpp) and guanosine-3', 5'-bis(diphosphate) (ppGpp), together called (p)ppGpp. These effector molecules are now emerging as master regulators for several physiological processes of bacteria including virulence, persistence, and antimicrobial resistance. (p)ppGpp may work independently or along with its cofactor DksA to modulate the activities of its prime target RNA polymerase and other metabolic enzymes, which are involved in different biosynthetic pathways. Enzymes involved in (p)ppGpp metabolisms are ubiquitously present in bacteria and categorized them into three classes, i.e., canonical (p)ppGpp synthetase (RelA), (p)ppGpp hydrolase/synthetase (SpoT/Rel/RSH), and small alarmone synthetases (SAS). While RelA gets activated in response to amino acid starvation, enzymes belonging to SpoT/Rel/RSH and SAS family can synthesize (p)ppGpp in response to glucose starvation and several other stress conditions. In this review, we will discuss about the current status of the following aspects: (i) diversity of (p)ppGpp biosynthetic enzymes among different bacterial species including enteropathogens, (ii) signals that modulate the activity of (p)ppGpp synthetase and hydrolase, (iii) effect of (p)ppGpp in the production of antibiotics, and (iv) role of (p)ppGpp in the emergence of antibiotic resistant pathogens. Emphasis has been given to the cholera pathogen *Vibrio cholerae* due to its sophisticated and complex (p)ppGpp metabolic pathways, rapid mutational rate, and acquisition of antimicrobial resistance determinants through horizontal gene transfer. Finally, we discuss the prospect of (p)ppGpp metabolic enzymes as potential targets for developing antibiotic adjuvants and tackling persistence of infections.

## Introduction

**Figure 1:**
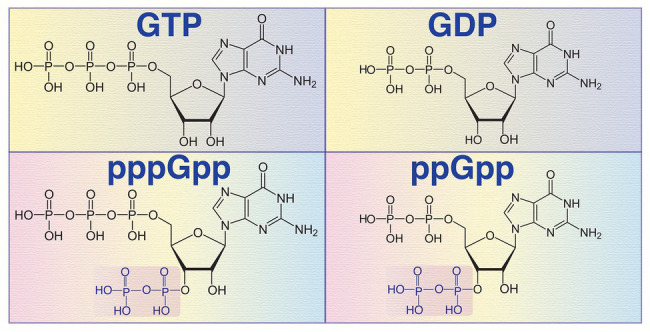
Chemical structures of guanosine triphosphate (GTP), guanosine diphosphate (GDP), guanosine pentaphosphate (pppGpp), and guanosine tetraphosphate (ppGpp) molecules. The pyrophosphate group of pppGpp and ppGpp at the 3' hydroxyl (OH) position is transferred by the (p)ppGpp synthetase from another purine nucleotide ATP.

Living organisms from three domains of life (bacteria, archaea, and eukarya) use a number of purine derivatives like guanosine pentaphosphate (pppGpp), guanosine tetraphosphate (ppGpp), cyclic di-GMP (c-di-GMP), etc., as intracellular signaling molecules. Bacteria use these small molecules to monitor intra- and extracellular environmental conditions and modulate their growth and multiplications in response to the availability of nutrients and related local cues (Potrykus and Cashel, 2008; Tozawa and Nomura, 2011; Hauryliuk et al., 2015). Over 50 years ago, Cashel identified the small molecule alarmones, pppGpp, and ppGpp (Figure 1), collectively known as (p)ppGpp, as the key players for the bacterial stringent response under nutrient limitations and other stressful conditions (Cashel, 1969). Later several studies revealed that the effector molecules of stringent response (p)ppGpp modulates bacterial multiplication rate and survival during nutrient limitations, exposure to antimicrobial compounds, xenobiotics, and osmotic stress (Hauryliuk et al., 2015; Irving and Corrigan, 2018). The alarmone (p)ppGpp is the derivatives of guanosine nucleosides, where guanosine triphosphate (GTP) pyrophosphokinases (RelA/SpoT/RSH/RelV/RelP/RelQ) transfer a pyrophosphate moiety from ATP to the 3'-OH position of GTP/guanosine diphosphate (GDP; Cashel, 1969; Gaca et al., 2015; Ronneau and Hallez, 2019). It has been shown that the intracellular level of (p)ppGpp is critical for modulation of different bacterial physiological processes mainly by regulating the activities of RNA polymerase (RNAP), DNA primase (DnaG), growth rate, and several other metabolic enzymes in *Escherichia coli* (Potrykus and Cashel, 2008; Potrykus et al., 2011; Hauryliuk et al., 2015; Zhang et al., 2018; Wang et al., 2019). In addition, (p)ppGpp also modulates bacterial growth and viability indirectly through depletion of cellular level of guanosine and adenosine nucleotides or by repressing transcription of genes required for active growth (Kriel et al., 2012). In nutrient rich growth condition, the basal cellular level of (p)ppGpp in *E. coli* is less than 0.2 mM (Mechold et al., 2013). Upon induction of stress, the level of (p)ppGpp may increase from 10 to 100-fold depending upon the type of stress and the enzymes involved in the biosynthesis of different biomolecules (Kalia et al., 2013). Elevated level of (p)ppGpp may work independently or synergistically with the transcriptional factor DksA, an RNAP binding small transcriptional factor (Paul et al., 2004). It was discovered earlier that the *dksA* gene product suppresses temperature-sensitive growth and filamentation of a *dnaK* deletion mutant of *E. coli* (Kang and Craig, 1990). Later, it has been established that both DksA and (p)ppGpp biosynthetic enzymes are crucial for the stringent response in Gram-negative bacteria since, Δ*dksA* and Δ*relA*Δ*spoT* mutants exhibit similar phenotypes (Gourse et al., 2018). In addition, overexpression of DksA can compensate the loss of (p)ppGpp in regulating *uspA*, *livJ*, and *rrnBP1* (Magnusson et al., 2007). However, synergistic functions are not universal. DksA and (p)ppGpp can work independently or may have opposite effects on one another. For example, *E. coli* Δ*dksA* cells aggregate more efficiently compared to its isogenic wild-type strain. Similarly, overexpression of DksA decreases the adhesion of wild-type cells. In contrast, *E. coli* Δ*relA*Δ*spoT* mutant called (p)ppGpp^0^ cells failed to sediment in a similar experimental condition and the adhesion phenotype is not affected upon overexpression of DksA (Magnusson et al., 2007). In addition, transcription of the *argX* operon containing *argX*, *hisR*, *leuT*, and *proM* genes is activated by DksA but inhibited in the presence of (p)ppGpp and DksA (Lyzen et al., 2016).

Other than the function in stringent response, (p)ppGpp also plays important roles in modulating bacterial virulence gene expression (Dalebroux et al., 2010; and the references therein), sporulation (Crawford and Shimkets, 2000), biofilm formation (He et al., 2012), antibiotic resistance (Wu et al., 2010; Strugeon et al., 2016), tolerance (Kim et al., 2018), and persistence (Hauryliuk et al., 2015; Harms et al., 2016). In order to access host cell nutrients, colonization on the cell surface and detachment from mucosal surface, pathogenic bacteria use (p)ppGpp signaling networks to modulate expression of genes those are part of secretion systems, flagellar components, adhesins, and serine/metallo proteases (Dalebroux et al., 2010; Pal et al., 2012; and reference therein). Regulation of spore formation in certain bacteria mediated by (p)ppGpp through complex array of regulatory circuits that sense the environmental signals through altered levels of intracellular (p)ppGpp leading to rapid change in the expression of relevant genes involved in spore formation (Crawford and Shimkets, 2000). It has been shown that the stringent response positively modulates biofilm formation in *E. coli*, *Vibrio cholerae*, and *Streptococcus mutans* (He et al., 2012; Teschler et al., 2015; Strugeon et al., 2016). In *Pseudomonas aeruginosa*, the antibiotic tolerance of nutrient-limited and biofilm dwelling cells is mediated by active responses to starvation where stringent response plays a crucial role (Nguyen et al., 2011). This starvation mediated protective mechanism in *P. aeruginosa* has been shown to be linked with tolerance under reduced level of oxidative stress in bacterial cells and, therefore, inactivating this protective mechanism sensitized biofilms by several orders of magnitude to different classes of antibiotics allowing enhanced efficacy of antibiotic treatment in experimental infection in an animal model (Nguyen et al., 2011).

From various studies, it appears that emergence of instant antibiotic resistant clones in a susceptible bacterial population solely depends on: (i) target modifications, (ii) reduced accessibility of antibiotics to the target, (iii) decreased effective concentration of antibiotic by reducing the membrane permeability or by increasing efflux activity, and (iv) acquisition of antibiotic resistance genes from other microbial species (Gaca et al., 2015; Verma et al., 2019; Das et al., 2020; Jung et al., 2020; Pant et al., 2020). A recent study has shown that the expression levels of ~300 and ~400 genes (total ~700 genes) are upregulated and downregulated, respectively, within 5 min upon induction of (p)ppGpp (Sanchez-Vazquez, 2018). In *Enterococcus faecalis* (p)ppGpp^0^ cells, it has been found that the genes and pathways involved in pyruvate production and heterolactic fermentation are induced (Gaca et al., 2013). More importantly, (p)ppGpp induced RpoS expression, the stress response sigma factor, may lead to overproduction of error prone DNA polymerase IV (Pol IV; Storvik and Foster, 2010). In addition to antibiotic resistance, (p)ppGpp also reduces efficacy of antibiotics by inducing antibiotic tolerant persister cell formation in Gram-negative and Gram-positive bacterial populations (Korch et al., 2003; Kaspy et al., 2013).

As of 5th August 2020, more than 27,936 articles and reports are available in the National Center for Biotechnology Information^1^ on the stringent response in bacteria. Considering this vast literature, however, the present review will focus on: (i) metabolisms, biosynthetic enzymes, signals, interaction partners, and targets of (p)ppGpp; (ii) effect of (p)ppGpp in antibiotic production; (iii) role of (p)ppGpp in antibiotic resistance in bacterial pathogens; and (iv) (p)ppGpp biosynthetic enzymes as potential targets for developing antibiotic adjuvants.

## Body

### (p)ppGpp Metabolism in Bacteria

**Figure 2:**
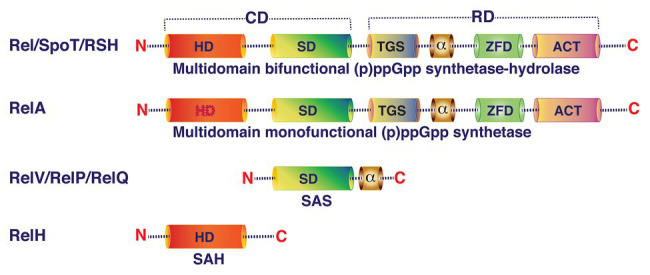
Schematic representation of the domain organization of (p)ppGpp synthetase/hydrolase family of proteins. Bifunctional long multidomain (p)ppGpp synthetase-hydrolase and monofunctional long multidomain (p)ppGpp synthetase enzymes are composed of two regions: (i) N-terminal catalytic domain (CD) and (ii) C-terminal regulatory domain (RD). Both synthetase and hydrolase domains (SD and HD, respectively) are functional in bifunctional enzymes (Rel/RSH/SpoT). In monofunctional multidomain, (p)ppGpp synthetase enzyme (RelA) hydrolase activity is lost due to accumulation of spontaneous mutations in the catalytic residues. The C-terminal regulatory region of the multidomain protein contains TGS domain (ThrRS, GTPase, and SpoT), conserved α helical domain, ZFD domain (zinc-finger or conserved cysteine domain), and ACT (aspartate kinase, chorismate, and TyrA) RNA recognition motif. The small alarmone synthetase (SAS) enzyme (RelV/RelP/RelQ) contains SD and a small oligomeric α-domain. The RelH/small alarmone hydrolase (SAH) contains only the HD.

**Figure 3:**
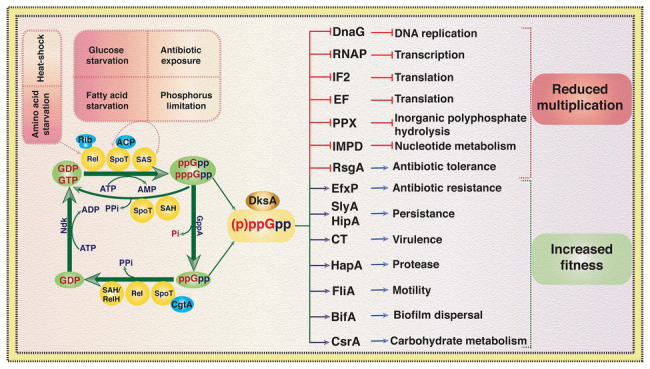
An overview of signaling, metabolism, and effect of (p)ppGpp in virulence, antibiotic resistance, and other cellular processes in bacteria. GDP and GTP are converted to (p)ppGpp during stress conditions or upon exposure to xenobiotics due to transfer of a pyrophosphate moiety from ATP to the 3'-OH position of ribosugar of guanosine nucleotide. GTP pyrophospho kinase activity of Rel, SpoT, and SAS can mediate the transfer of pyrophosphate to GTP or GDP depending on the stress signaling and activity of other cellular proteins like acyl carrier protein (ACP) and functional state of protein synthesis machinery, i.e., ribosome (Rib). pppGpp can be converted to ppGpp due to phosphatase activity of GppA. SpoT, Rel, and SAH can hydrolyze the pyrophosphate moiety from 3'-OH position of pppGpp and ppGpp and generate GTP or GDP, respectively. Guanine nucleotide binding protein of the Obg/GTP1 subfamily CgtA induces (p)ppGpp hydrolase activity of SpoT. Nucleoside diphosphate kinase (Ndk) converts GDP to GTP using ATP as phosphate donor. Elevated intracellular level of (p)ppGpp independently or in conjunction with transcriptional factor DksA regulates several cellular processes including DNA replication, transcription, protein biosynthesis, carbohydrate metabolism, virulence, motility, antibiotic resistance, and biofilms formation. (p)ppGpp regulates several proteins involved in nucleotide metabolism, persistence, antibiotic resistance, etc., however, for simplicity, a representative protein for each case is depicted here and more details can be found in the references cited. SAS, small alarmone synthase; SAH, small alarmone hydrolase; GppA, guanosine-5'-triphosphate, 3'-diphosphate pyrophosphatase; DnaG, DNA primase; RNAP, RNA polymerase; IF2, initiation factor 2; EF, elongation factor; PPX, exopolyphosphatase; IMPD, inosine monophosphate dehydrogenase; RsgA, small ribosomal subunit biogenesis GTPase; EfxP, efflux pumps; SlyA, transcriptional regulator of *Salmonella enterica*; HipA, toxin component of *Escherichia coli* TA module; CT, cholera toxin; HapA, hemagglutinin protease; FliA, flagella regulatory sigma factor; BifA, biofilm formation protein; CsrA, carbon storage regulator (Dalebroux et al., 2010; Ronneau and Hallez, 2019; and the references therein).

Principally, (p)ppGpp homeostasis in bacterial cells depends on the availability and activity of the four classes of enzymes: (i) multi-domain bifunctional (p)ppGpp synthetase-hydrolase, (ii) multi-domain monofunctional (p)ppGpp synthetase, (iii) short alarmone synthetase (SAS), and (iv) short alarmone hydrolase (SAH), also called RelH (Figure 2). Bifunctional (p)ppGpp synthetase-hydrolase enzymes (Rel/RSH/SpoT) can modulate their conformation depending on the environmental conditions with the help of their regulatory domains and stimulate their GTP/GDP pyrophosphokinase or pyrophosphohydrolase activities to synthesize or hydrolyze (p)ppGpp, respectively (Ronneau and Hallez, 2019). In the presence of (p)ppGpp synthetase, hydrolase activity of the bifunctional enzyme is essential for viability (Xiao et al., 1991; Das and Bhadra, 2008). However, bacterial cells can survive in the absence of (p)ppGpp synthetase activity in nutrient rich environments but it is essential for viability in nutrient limited medium (Xiao et al., 1991). Enzymes with (p)ppGpp synthetase-hydrolase activities are widely conserved across bacterial phyla (Atkinson et al., 2011). Nevertheless, presence of (p)ppGpp synthetase-hydrolase domain containing proteins has also been reported in eukarya including *Arabidopsis thaliana*, *Nicotiana tabacum*, and *Chlamydomonas reinhardtii* (Sun et al., 2010; Ito et al., 2012). In Firmicutes, (p)ppGpp synthetase-hydrolase domain containing enzymes are the major source of stringent response effector molecules during amino acid, glucose, and fatty acid starvations (Wolz et al., 2010). In contrast, several Proteobacteria use the bifunctional (p)ppGpp synthetase-hydrolase enzyme for alarmone synthesis during glucose and fatty acid starvations but not during amino acid starvation (Xiao et al., 1991; Cashel, 1969). Upon sensing nutrient depletions or other stress conditions, Rel/RSH/SpoT catalyze pyrophosphorylation of GDP or GTP at the 3' hydroxyl (OH) position using ATP as pyrophosphate donor (Figure 3). Once reprogramming of cellular functions from vegetative to survival mode is accomplished with the help of elevated intracellular levels of (p)ppGpp, the bifunctional Rel/RSH enzyme then may change its conformation from (p)ppGpp synthetase-ON/hydrolase-OFF to (p)ppGpp hydrolase-ON/synthetase-OFF state to reduce the (p)ppGpp level to restore again the gene expression and metabolic functions of enzymes associated with growth and multiplication under favorable conditions (Hogg et al., 2004). Although the exact molecular mechanism is unknown, but it has been proposed that the regulatory domains located at the carboxy terminal domain of Rel/RSH/SpoT play important roles in the modulation of synthetase and hydrolase functions of the bifunctional (p)ppGpp synthetase-hydrolase enzymes (Angelini et al., 2012).

Monofunctional (p)ppGpp synthetase, the enzyme that synthesizes (p)ppGpp only, further subdivided into two major sub-classes based on their size and domain organization: (i) long multi-domain monofunctional (p)ppGpp synthetase and (ii) short monodomain monofunctional (p)ppGpp synthetase also known as SAS (Figure 2). Presence of multi-domain monofunctional (p)ppGpp synthetase (for example, RelA) is common in Proteobacteria. RelA is a ribosome-associated protein, which recognizes stalled ribosomes due to presence of uncharged tRNA in the A-site of ribosome during amino acid starvation. It has been reported that the large subunit ribosomal protein L11 is crucial for the activation of (p)ppGpp synthetase activity of RelA (Parker et al., 1976; Cashel et al., 1996). Wendrich et al. (2002) initially proposed a “hopping model” for RelA mediated (p)ppGpp synthesis during amino acid starved condition. According to this model the RelA molecule hops from ribosome to ribosome to synthesize one (p)ppGpp molecule per dissociation event. Later, English et al. (2011) using single-molecule tracking methodology proposed an “extended hopping” model for RelA mediated (p)ppGpp synthesis during amino acid starvation. According to the extended hopping model several molecules of (p)ppGpp are synthesized by the free but enzymatically active RelA upon its dissociation from ribosomes. However, findings of Li et al. (2016) contradict the extended hopping model and they proposed a “short hopping time” model for RelA mediated (p)ppGpp synthesis during amino acid starvation. According to this model, RelA synthesizes (p)ppGpp while bound to the 70S ribosomes. Currently, however, the reasons behind these differences are not clear. Although amino acid starvation is the major cause of activation of (p)ppGpp synthetase function of RelA, heat shock can also induce its synthetase activity in *E. coli* (Gallant et al., 1977; English et al., 2011). Short monodomain monofunctional (p)ppGpp synthetases are widely distributed among Firmicutes (*Bacillus subtilis*, *S. mutans*, *E. faecalis*, etc.), Actinobacteria (*Mycobacterium smegmatis*), Proteobacteria (*V. cholerae*), Archaea (*Methanosarcina acetivorans*), and Eukarya (*Dictyostelium discoideum*) (Lemos et al., 2007; Nanamiya et al., 2008; Das et al., 2009; Atkinson et al., 2011; Murdeshwar and Chatterji, 2012; and the references therein). Unlike RelA, the SAS sub-class can recognize glucose, fatty acids, and other starvations and catalyze (p)ppGpp synthesis by transferring a pyrophosphate moiety from ATP to the 3'-OH position of GTP or GDP probably following same mechanistic pathways like RelA/RSH/Rel enzymes (Figure 3). Since SAS enzymes are devoid of any additional regulatory domain, their regulation for (p)ppGpp synthesis activity may primarily be dependent at the gene expression level (Dasgupta et al., 2014; Ronneau and Hallez, 2019) and needs further investigation.

The monodomain monofunctional (p)ppGpp hydrolase, also known as small alarmone hydrolase (SAH), was initially identified and functionally characterized in the metazoa as an ortholog of bacterial SpoT (Sun et al., 2010). Like multi-domain bacterial (p)ppGpp hydrolases, the metazoan ortholog, called Mesh1, contains an active site for (p)ppGpp hydrolysis and it carries the conserved His-Asp box motif for binding with Mn^2+^. Later *in silico* analyses of more than 1,000 genome sequences identified seven subgroups of SAH (Atkinson et al., 2011). Recently, an SAH from *Corynebacterium glutamicum* has functionally been characterized and designated RelH (Ruwe et al., 2018). However, the regulatory signals, interaction partners, and importance of SAH in either kingdom are not clear. Thus, more intense research on these aspects of SAH is needed.

### Mechanism of Actions of (p)ppGpp

Accumulation of (p)ppGpp in the cytosol due to nutrient limitations or other stress conditions leads to change in bacterial cellular physiology by: (i) reprogramming of transcription of rRNA operons, ribosomal protein encoding genes, and others by regulating RNAP activity, (ii) stalling DNA replication by inhibiting DnaG, and (iii) modulating metabolic pathways by regulating activity of the associated enzymes (Figure 3). Single or multiple molecules of (p)ppGpp binds to its targets and modulate their activity independently or synergistically with its functional partner DksA. In *E. coli*, (p)ppGpp binds to at least two sites of RNAP enzyme (Ross et al., 2013). Crystal structure of RNAP holoenzyme showed that (p)ppGpp binds to the cleft surrounded by the α, β', and ω subunits (site 1) and also at an interface of RNAP and DksA (site 2). Binding of (p)ppGpp induces allosteric changes in RNAP, which either affects its catalytic activity by modulating the Mg^+2^ nucleotidyl transfer efficacy or by reducing stability of RNAP-promoter complex (Kanjee et al., 2012). DksA binds to the secondary channel of RNAP and can potentiate the effects (p)ppGpp by modulating RNAP-promoter complex stability (Perederina et al., 2004). In *B. subtilis*, (p)ppGpp regulates rRNA and ribosomal protein encoding genes transcription by interfering (p)ppGpp homeostasis. Elevated levels of (p)ppGpp inhibit hypoxanthine phosphoribosyl transferase (HprT) and GMP kinase (Gmk) activity in Firmicutes and reduce the intracellular GTP pool (Figure 3). HprT is essential for the conversion of hypoxanthine to IMP and guanine to GMP, while Gmk catalyzes synthesis of GDP from GMP. Both the enzymes are crucial for GTP biosynthesis in *B. subtilis* and other Firmicutes (Denapoli et al., 2013). It has been shown recently that in Gram-positive bacteria (p)ppGpp may bind with several ribosome-associated GTPases like RsgA, RbgA, Era, and HflX leading to strong inhibition of their activities (Corrigan et al., 2016). More recently, Zhang et al. (2018) have also reported similar target proteins, namely, RsgA, Era, HflX, etc., of (p)ppGpp in *E. coli*. However, the exact biochemical mechanism by which (p)ppGpp can inhibit GTPase activity is yet to be fully elucidated. Nonetheless, the findings clearly point toward the critical roles of (p)ppGpp in controlling ribosomal assembly/biogenesis in bacterial stress survival.

It is well established that (p)ppGpp drastically reduces bacterial multiplication by inhibiting initiation and elongation of DNA replication (Wang et al., 2007). It has been shown that (p)ppGpp binds to the RNA primer biosynthesis enzyme primase (DnaG) and directly inhibits its primer biosynthesis activity, which is essential for initiation of DNA replication (Figure 3). Initial finding hypothesized that the effect of (p)ppGpp in DNA replication is mostly restricted at the *oriC* region during initiation of DNA replication (Gourse and Keck, 2007). However, subsequent studies demonstrated that the arrest of DNA replication occurs throughout the chromosome during stringent response. Although elevated levels (>10–20-fold) of (p)ppGpp is detrimental to bacterial growth, complete lack of (p)ppGpp, called (p)ppGpp^0^ phenotype, render several bacterial species auxotrophic to different amino acids, defective in cell division, deficient in protease production, and poor survival upon exposure to xenobiotics (Hauryliuk et al., 2015). More importantly, increasing numbers of reports suggest that elevated level of (p)ppGpp reduces efficacy of several clinically important antibiotics, while (p)ppGpp^0^ strains are susceptible to numerous antibiotics with reduced minimum inhibitory concentration (MIC; Gaca et al., 2013; Kamarthapu et al., 2016; Hobbs and Boraston, 2019 and the references therein).

### Effect of (p)ppGpp in the Production of Antibiotics

Production of antibiotics in Actinobacteria, one of the most diverse groups of Gram-positive bacteria that produce most of the clinically used antibiotics, is influenced by the availability of nutrients and external signals. Such factors work either by modulating the expression level of antibiotic biosynthetic gene clusters or through pleiotropic regulators, which play important roles in bacterial intracellular signaling (van der Heul et al., 2018). Role of (p)ppGpp in antibiotic production has been demonstrated both in environmental isolates as well as in clinically important bacterial pathogens (Ochi, 1987; Jin et al., 2004; Gomez-Escribano et al., 2008; van der Heul et al., 2018). Different soil living Gram-positive bacteria including *B. subtilis*, *Streptomyces clavuligerus*, produce a variety of antibiotics to inhibit the growth of other bacteria living in the same environment (van der Meij et al., 2017; and the references therein). Various reports suggest that (p)ppGpp may either positively or negatively regulate antibiotic production in Actinobacteria. For example, *Streptomyces coelicolor* synthesizes two well-known antibiotics, the polyketide actinorhodin, and the tripyrolle undecylprodigiosin and the production of each them was decreased in the Δ*relA* mutant of *S. coelicolor* under a continuous culture condition (Kang et al., 1998). On the other hand, it has been shown in *S. clavuligerus* that (p)ppGpp negatively regulates the production of clavulanic acid and cephamycin C antibiotics in the absence of (p)ppGpp biosynthetic gene *relA* (Gomez-Escribano et al., 2008). Role of (p)ppGpp in the antibiotic production has also been demonstrated in other species of *Streptomyces* including *Streptomyces antibioticus* and *Streptomyces griseus* (Sivapragasam and Grove, 2019). (p)ppGpp may promote antibiotic production in these bacteria by inducing transcription of antibiotic biosynthetic gene clusters. In fact, it has been shown that the elevated level of intracellular (p)ppGpp in *S. coelicolor* leads to reduction in the expression of vegetative sigma factor and induction of expression of alternative sigma factor, which play important role in the transcription of antibiotic biosynthesis gene cluster (Hesketh et al., 2007).

### Bacterial Antibiotic Resistance and (p)ppGpp

**Figure 4:**
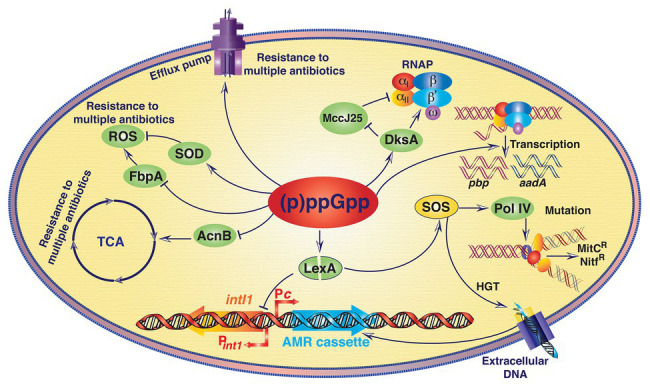
A schematic representation showing the (p)ppGpp mediated antimicrobial resistance (AMR) in bacteria. Except AMR cassette acquisition at the integron integrase locus all other processes that are directly or indirectly associated with antibiotic resistance due to increased level of (p)ppGpp are non-inheritable. (p)ppGpp induces antibiotic resistance in bacteria *via* distinct mechanisms (see details in text), which are as follows. (i) (p)ppGpp increases the efficiency of horizontal gene transfer (HGT) and AMR cassette acquisition by derepressing the integron integrase 1 (*intI1*) gene promoter (P*_intI1_*). Elevated level of (p)ppGpp induces autoproteolytic activity of SOS response master regulator LexA, and thus increases expression of the *intI1* gene. In addition, SOS induction also derepressed the expression of error-prone DNA polymerase IV (Pol IV) and upturns accumulation of spontaneous point mutations *via* DNA replication, which in turn escalates development of resistant variants. (ii) (p)ppGpp upregulates the expression of the aminoglycoside adenylyl transferase gene (*aadA*), penicillin binding protein coding gene (*pbp*), and different components of the efflux pumps. (iii) (p)ppGpp helps DksA to compete with microcin J25 (MccJ25) peptide antibiotic for binding with RNA polymerase (RNAP) and confers resistance against this peptide antibiotic. (iv) (p)ppGpp represses the expression of aconitase B (AcnB) and suppresses tricarboxylic acid (TCA) cycle and increases non-inheritable resistance to antibiotics. (v) (p)ppGpp represses the expression of Fe(III) ABC transporter substrate-binding protein (FbpA) and activates superoxide dismutase (SOD) activity. Both the modulations reduce the level of reactive oxygen species (ROS) leading to increase in resistance against tetracycline and other antibiotics. Solid arrows indicate activating interactions and T-arrows indicate inhibiting interactions. Bent arrows at the integron locus indicate promoter for AMR cassette (P*c*) and P*_intI1_*. MitC^R^, Mitomycin C resistance; Nitf^R^, Nitrofurantoin resistance.

Antibiotics, natural or synthetic chemical compounds that interfere with microbial growth or eliminate microbes from their niche, are one of the most important drugs in the medical history that has ever been discovered and deployed to prevent or cure microbial infections (Fischbach and Walsh, 2009). However, emergence of antibiotic resistance (AMR) and rapid dissemination of resistant traits in the clinically important bacterial species has drastically reduced the efficacy of several therapeutic agents commonly used to prevent or treat microbial infection (Das et al., 2017). AMR could be intrinsic or acquired and a wide range of metabolic pathways can confer resistance phenotype in bacteria (Figure 4; Das et al., 2020). Horizontal gene transfer (HGT), *de novo* mutation and changes in the expression profile of secreted proteins determine the rate of emergence of resistant variants (Davies and Davies, 2010). Alarmone molecule (p)ppGpp induces HGT while bacterial species are living in biofilms (Strugeon et al., 2016). Elevated level of (p)ppGpp derepressed the *intI1* promoter (P*_intI1_*) by inducing autoproteolytic activity of SOS response master regulator LexA, possibly through reducing the activity of exopolyphosphatase enzyme PPX (Strugeon et al., 2016). In normal physiological conditions, LexA dimer reduces the expression of IntI1 integrase by inhibiting P*_intI1_*. Increased expression of integrin integrase due to (p)ppGpp mediated moderate increase of SOS response in biofilm environment helps integron mediated acquisition/exchange of antibiotic resistance gene cassettes through HGT (Figure 4).

It has been observed that bacterial (p)ppGpp^0^ strains are usually sensitive to diverse xenobiotics, antibiotics, and ultraviolet radiation (Hobbs and Boraston, 2019; and the references therein). Bacterial population that does not carry any specific antibiotic resistance trait or altered target, a small fraction of this population of cells may develop persistence to tolerate lethal doses of antibiotics, which seems to be mediated by the elevated level of intracellular (p)ppGpp. From several recent studies, it is gradually becoming clear that the increased intracellular levels of (p)ppGpp most likely activates the bacterial toxin-antitoxin module, also called TA module, through a complex regulatory mechanism, and the released toxin moiety then helps in maintaining the high intracellular levels of (p)ppGpp and thus, leading to the genesis of persister cells (Korch et al., 2003; Kaspy et al., 2013; Harms et al., 2016). It has been demonstrated recently that (p)ppGpp may play an important role in the nucleotide excision DNA repair process. Absence of (p)ppGpp or its functional partner DksA render bacterial cells highly sensitive to mitomycin C, 4-nitroquinoline 1-oxide and nitrofurantoin, the antibiotics that kill bacterial species by interfering DNA or RNA synthesis (Kamarthapu and Nudler, 2015). Bacterial cells with higher basal level of (p)ppGpp are more resistant to antibiotics that work through interfering nucleic acid biosynthesis or damage. (p)ppGpp directly modulates the activity of RNAP, therefore, it could support UvrD for the repair of damaged DNA through transcription coupled DNA repair pathway. In addition, induction of SOS during stress conditions including exposure to sub-lethal concentration of antibiotics also derepressed the expression of error-prone DNA polymerase IV and increases accumulation of spontaneous mutations, which in turn escalate development of resistant variants (Layton and Foster, 2005). When *Salmonella enterica* cells are treated with aminoglycoside antibiotics, they showed (p)ppGpp mediated upregulation of expression of the aminoglycoside adenylyl transferase gene (*aadA*) and thus, resulting in resistance to streptomycin and spectinomycin antibiotics (Koskiniemi et al., 2011).

Increased basal level of (p)ppGpp also contributes in the emergence of antibiotic resistant bacterial cells by directly modulating the expression profile of genes encoding penicillin binding proteins (PBPs) or components of the efflux pumps (Aedo and Tomasz, 2016). Rodionov and Ishiguro (1995) have reported about the importance of (p)ppGpp in inducing resistance against beta-lactam antibiotics namely penicillin. They showed that controlled expression of the *relA* gene resulted in inhibition of peptidoglycan and phospholipid biosynthesis subsequently resulting in penicillin tolerance. Oxacillin resistance in *Staphylococcus aureus* is directly associated with increased (p)ppGpp synthetase activity of RelQ or decreasing (p)ppGpp hydrolase activity of the Rel enzyme (Mwangi et al., 2013). Similarly, increase in intracellular (p)ppGpp concentration in *E. faecalis* has been found to be responsible for the development of non-inheritable resistance against the antibiotic vancomycin (Abranches et al., 2009). Very recently it has been shown that the *rel* gene deleted *Mycobacterium tuberculosis* cells become more susceptible to isoniazid killing under nutrient starved condition and also in the lungs of infected mice (Dutta et al., 2019). Thus, it appears that inhibition of Rel may be a useful approach to eliminate *M. tuberculosis* persister cells. On the other hand, a direct correlation has been found between intracellular (p)ppGpp accumulation and increase in resistance against the peptide antibiotic microcin in *E. coli* (Pomares et al., 2008). It was hypothesized that (p)ppGpp helps DksA to compete with microcin J25 peptide antibiotic for binding with RNAP (Figure 4). A very recent study showed that (p)ppGpp induces the expression of efflux pump related genes in *Acinetobacter baumannii* (Jung et al., 2020). MIC of several antibiotics drastically reduced in (p)ppGpp^0^ *A. baumannii* strain compared to its wild-type ancestor, possibly due to reduction in expression of antibiotics expelling efflux pumps (Jung et al., 2020). In *E. coli*, homeostasis of purine nucleosides is regulated by the nucleosidase PpnN by cleaving nucleoside monophosphates. (p)ppGpp binds to the PpnN and stimulates its catalytic activity, which helps cells to tolerate increased concentration of antibiotics (Zhang et al., 2019). The most common antibiotics for the treatment of cholera patients are tetracycline, erythromycin, and chloramphenicol. A recent study has shown that the increased level of intracellular (p)ppGpp helps *V. cholerae* cells to reduce the efficacy of all these antibiotics, at least under the laboratory testing conditions (Kim et al., 2018). (p)ppGpp metabolic cycle in *V. cholerae* is fairly unique in comparison to other Gram-negative bacteria (Das et al., 2009). The alarmone helps cholera pathogens for survival, disease development, and antibiotic resistance. Wild-type *V. cholerae* strain and its (p)ppGpp overproducing isogenic mutant Δ*relA*Δ*spoT* can survive better in the presence of different antibiotics compared to (p)ppGpp° Δ*relA*Δ*spoT*Δ*relV* strain. It was hypothesized that (p)ppGpp suppresses tricarboxylic acid cycle by repressing the aconitase B encoding gene *acnB* and increases non-inheritable resistance to antibiotics (Kim et al., 2018). In addition, (p)ppGpp also represses the expression of iron (III) ABC transporter substrate-binding protein in the wild-type *V. cholerae* strain, which is important for the generation of reactive oxygen species (ROS) and increased susceptibility to tetracycline (Figure 4). Nevertheless, it has also been reported that the Δ*dksA V. cholerae* mutants are also highly sensitive to different antibiotics compared to isogenic wild-type strain (Kim et al., 2018). In a murine infection model, substantial increase in the efficacy of several fluoroquinolones against the (p)ppGpp^0^ *P. aeruginosa* mutant strain has been noticed compared to that of isogenic wild-type strain (Nguyen et al., 2011). In fact, it has been observed that increased intracellular concentration of (p)ppGpp may modulate non-inheritable resistance to different antibiotics possibly through regulation of bacterial ROS production. Several studies have demonstrated that the high intracellular levels of (p)ppGpp is associated with the antioxidant defense mechanisms in *P. aeruginosa* (Nguyen et al., 2011; Martins et al., 2018). Superoxide dismutase (SOD), an enzyme that catalyzes the dismutation of the superoxide (O_2_^−^) radical into ordinary molecular oxygen (O_2_) and hydrogen peroxide (H_2_O_2_), is a key enzyme involved in stringent response mediated drug resistance in stationary phase cells of *P. aeruginosa* (Martins et al., 2018). It has been shown that deletions of (p)ppGpp biosynthetic genes reduce the SOD activity and diminish drug tolerance phenotypes in stationary phase cells of *P. aeruginosa*. Complementation of SOD activity in the (p)ppGpp^0^ *P. aeruginosa* was able to restore the non-inheritable resistance against several antibiotics almost similar to that of isogenic wild-type strain. The authors have also found that in the absence of SOD activity, the membrane permeability of *P. aeruginosa* cells is high, which leads to increase in drug internalization and thus raising the efficacy of antibiotics.

### Stringent Response Inhibitors Are Potential Antibiotic Adjuvant

The conserved distribution of stringent response across the bacterial phyla and its important roles in developing persistent bacterial infections made it a promising drug target. Subpopulation of bacteria can survive upon exposure to lethal concentration of antibiotic through growth arrest, while others are not (Balaban et al., 2004). Persister bacteria exhibit non-inheritable resistance to the bactericidal antibiotics (Dutta et al., 2019). It is well known that the stringent response effector molecules (p)ppGpp plays an important role in fine-tuning bacterial growth during nutritional stress and exposure to antibiotics. It helps bacterial pathogens to survive during growth-limiting conditions, to increase non-inheritable resistance and to develop persister cells for prolong infection (Abranches et al., 2009; Dutta et al., 2019). The major bacterial persistent infections are usually caused by *M. tuberculosis*, *Helicobacter pylori*, *Salmonella* Typhi, and several others, where the pathogens can overcome the antimicrobial effect of routinely used antimicrobials (Kester and Fortune, 2014). As discussed above, several studies have indicated that the stringent response plays a pivotal role in developing non-inheritable resistance and persister cell formation. Therefore, in recent years’ attempts have been made to develop stringent response inhibitors to block long-term persistence, sporulation, and biofilm formation with a hope that blocking such important cellular functions may help in increasing the efficacy of antimicrobial drugs (Wexselblatt et al., 2012, 2013; Syal et al., 2017a,b). Thus, a number of potential stringent response inhibitory molecules, namely, 2'-deoxyguanosine-3'-5'-di(methylene bisphosphonate), relacin, relacin analog 2d, vitamin C, etc., have been developed and shown to be effective against *B. subtilis*, *Bacillus anthracis*, *M. smegmatis*, and *E. coli* both under *in vitro* and *in vivo* growth conditions (Wexselblatt et al., 2012, 2013; Syal et al., 2017a,b). Relacin, a well-studied ppGpp analog in which 3'-pyrophosphate moieties were substituted with glycyl-glycine dipeptide, inhibits (p)ppGpp synthetic activity of Rel/RelA proteins of both Gram-positive and Gram-negative bacteria under *in vitro* conditions. It has been demonstrated that relacin reduces bacterial viability by preventing the cells entering into stationary growth phase (Wexselblatt et al., 2012). Thus, it seems that inhibiting the (p)ppGpp synthesis activity in pathogens by stringent response inhibitor is a potential approach to reduce viability of persister and shortening the duration of antibiotic treatment of a patient. Development of such promising inhibitors against RelA/SpoT/RSH enzymes and its use as antibiotic adjuvants can potentially help in eradicating several chronic bacterial infections.

## Conclusion

Although the (p)ppGpp metabolic pathway was originally discovered in bacteria about 50 years ago, continued research on this important molecule has made it clear that these alarmones are widely synthesized by the living organisms from all three domains of life. Enzymes involved in the (p)ppGpp metabolism and signals that activate or repress stringent response share substantial similarity between different organisms. Overwhelming evidences established that other than nutrient limitations, antibiotics, and non-antibiotic xenobiotics activate stringent response and help microbes to survive even in the absence of specific resistance genes. In addition to the regulation of DNA replication, RNA transcription and protein synthesis, (p)ppGpp molecules have a wide range of heterogeneous targets depending on the organisms and their habitats. It is becoming clear that the modulations of the activities of several such targets/proteins are linked with the non-heritable resistance to antibiotics. The genetic program linked with the bacterial antibiotic resistance in the absence of specific resistance genes appears to have multiple components. Activity of several such molecules intricately depends on the alarmone (p)ppGpp and other small signaling molecules, which warrants further in depth research for effective management of infectious diseases.

## Author Contributions

BD and RKB designed the review, done literature search, and wrote the manuscript. All authors contributed to the article and approved the submitted version.

### Conflict of Interest

The authors declare that the research was conducted in the absence of any commercial or financial relationships that could be construed as a potential conflict of interest.

## Footnotes

^1^https://www.ncbi.nlm.nih.gov/
